# Soil-transmitted helminthiasis (STH) endemicity and performance of preventive chemotherapy intervention programme in Nigeria (in year 2021)

**DOI:** 10.1038/s41598-023-37402-8

**Published:** 2023-06-22

**Authors:** Oyetunde T. Oyeyemi, Oluyemi A. Okunlola

**Affiliations:** 1 Department of Biosciences and Biotechnology, University of Medical Sciences, Ondo City, Ondo State Nigeria; 2 Department of Mathematical and Computer Sciences, University of Medical Sciences, Ondo City, Ondo State Nigeria

**Keywords:** Diseases, Health care

## Abstract

Preventive chemotherapy (PC) is an important tool to address transmission and reduce morbidities associated with soil-transmitted helminths (STHs). The aim of the study is to assess the PC implementation programme coverage and relate the same to the endemicity of STH in Nigeria. The secondary data collected by the World Health Organization (WHO) through the expanded special project for elimination of neglected tropical diseases (ESPEN) and made available at the ESPEN portal was used for the study. The PC implementation coverage and frequency of treatment were evaluated and related to STH endemicity levels in Nigeria. STH was actively transmitted in all six geopolitical zones of Nigeria. The southern part of Nigeria was more endemic compared with northern Nigeria. There was no PC intervention in preschoolers and effective PC coverage (19.3%) fell below the WHO ≥ 75% PC coverage index benchmark in school children. The percentages of children that harbour low, moderate, and high STH infections were 41.5, 41.6, and 13.4%, respectively. Multiple treatments did not necessarily reduce the endemicity of STH on certain occasions. STH continues to be a public health threat in Nigeria. The current treatment strategies should be reviewed to accommodate preschoolers in PC implementation programmes. Treatment should be integrated with the WASH programme in order to achieve a lasting impact.

## Introduction

Soil-transmitted helminthiases (STH) are among the most widely transmitted neglected tropical diseases affecting the world's poorest and most deprived communities. Over 1.5 billion people are infected with soil-transmitted helminths (STHs) worldwide and more than 800 million children are at risk of infection, and therefore, would need treatment and preventive interventions^1^. According to the World Health Organisation, approximately 1.5 billion individuals worldwide are believed to be infected with at least one species of STHs, leading to the loss of approximately 5 million disability-adjusted life years (DALYs)^2,3^. Moreover, severe morbidity associated with STHs affects around 300 million people, resulting in an estimated annual death toll ranging from 12,000 to 135,000^4^. In developing countries, the disease has significant health and socio-economic repercussions^5^. STHs are disproportionately prevalent in many countries of sub-Saharan Africa, especially Nigeria, the Democratic Republic of Congo, Ethiopia, Cameroon, Angola, Mozambique, Madagascar, Equatorial Guinea, and Gabon, where moderate-to-heavy intensity infections predominate^6^.

STHs control has achieved significant progress over the past decade as a result of increased collaboration, country commitment, and donors support. At this time, the WHO 2020 Neglected Tropical Diseases Roadmap target has been to provide regular anthelmintic treatment to at least 75% of children aged 5–14 years living in settings with infection prevalence over 20%. Sub-Saharan Africa nearly achieved its goal of deworming 70% of at-risk children by 2018^6^. It is, however, difficult to conclude that the recent commitment to STH control marked by an increase in preventive chemotherapy coverage has culminated in a corresponding decrease in the endemicity of STH in sub-Saharan African countries. For example, the previous interventions produced little or no results in several sub-Saharan countries despite the general global decline recorded in STH incidence, especially in the Americas and Asia^7^. Evidence has shown that where the decline in STH prevalence was recorded, such was strongly facilitated by socioeconomic development and integration of water, sanitation, and hygiene (WASH) interventions^8,9^.

Nigeria has a long history of STH endemicity with prevalence ranging from low-to-high worm burden recorded in all the states of the country^10^. Nigeria has four main species of STHs: *Ascaris lumbricoides*, *Trichuris trichiura*, *Ancylostoma duodenale*, and *Necator americanus*. The transmission of these infections occurs through the presence of eggs in human faeces, which subsequently contaminate the soil in regions characterised by inadequate sanitation practices. In children, STH infection is estimated to cause anaemia, vitamin A deficiency, malnutrition, loss of appetite, retarded growth, and reduced learning abilities^11^.

There have been consistent reports on the incidence of STH across the six geopolitical zone of Nigeria despite the recently renewed efforts to significantly abate STH transmission. The common challenges are the sporadic and poorly coordinated interventions, and the non-blending of preventive chemotherapy-related interventions with other interventions such as WASH. This is more worrisome as improved advocacies for sanitation, health education, and targeted chemotherapy have not produced a corresponding decrease in the disease burden in many high-risk communities in Nigeria^12–14^. Inability to implement interventions in security-challenged areas of Nigeria is another factor undermining the control effort^15^.

We hypothesised that effective preventive chemotherapy would reduce STH endemicity in Nigeria. So, this study aimed to evaluate the impact of preventive chemotherapy on the endemicity of STH in Nigeria. The study will give an objective assessment of the overall success of STH preventive chemotherapy programme in the country with a view to providing recommendations for programme implementation improvement.

## Results

**Figure 1:**
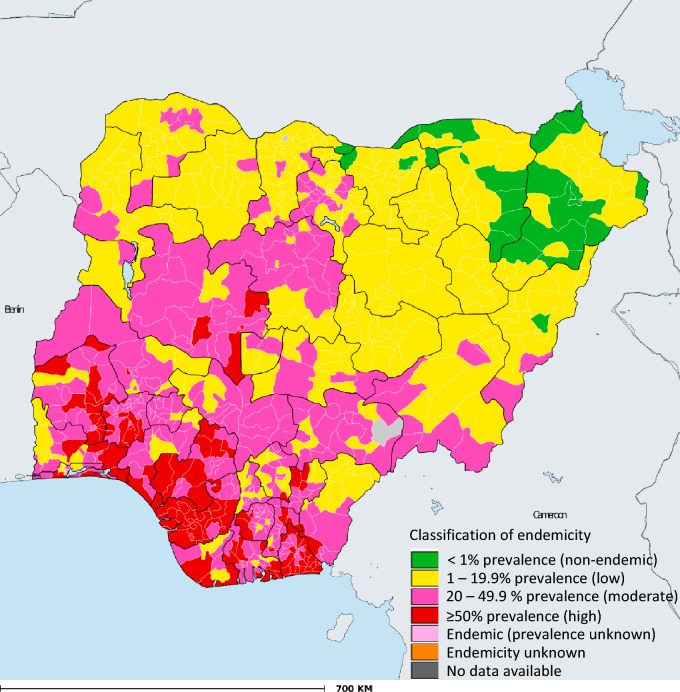
Status of STH in Nigerian children—2021. *Data Source*: Data provided by health ministries to ESPEN through WHO reporting processes. All reasonable precautions have been taken to verify this information.

The distribution of STH infection among children in Nigeria is presented in Fig. 1. Generally, southern Nigeria (South-west, South-east, and South-south regions) showed higher STH endemicity compared to zones in northern Nigeria. Our study revealed that STH is transmitted in all of Nigeria's geopolitical zones. Six southern states including; three Southwestern states (Ogun, Oyo, and Osun), and three South-South states (Edo, Delta, and Bayelsa) are the most endemic regions in Nigeria as moderate and high STH infections were often reported in the regions. Most states in Southeastern Nigeria including; Imo, Abia, and Ebonyi, and two Southwestern states, Ondo and Ekiti, were moderately endemic for STH infections. Similarly, the North-central states such as Kwara, Benue, Kogi, and Niger showed predominant moderate STH infections. Most of the states in the North-east (Bauchi, Yobe, Adamawa, and Taraba) and North-west (Sokoto, Katsina, Kano, and Jigawa) recorded low or light STH infections (Fig. 1).

**Figure 2:**
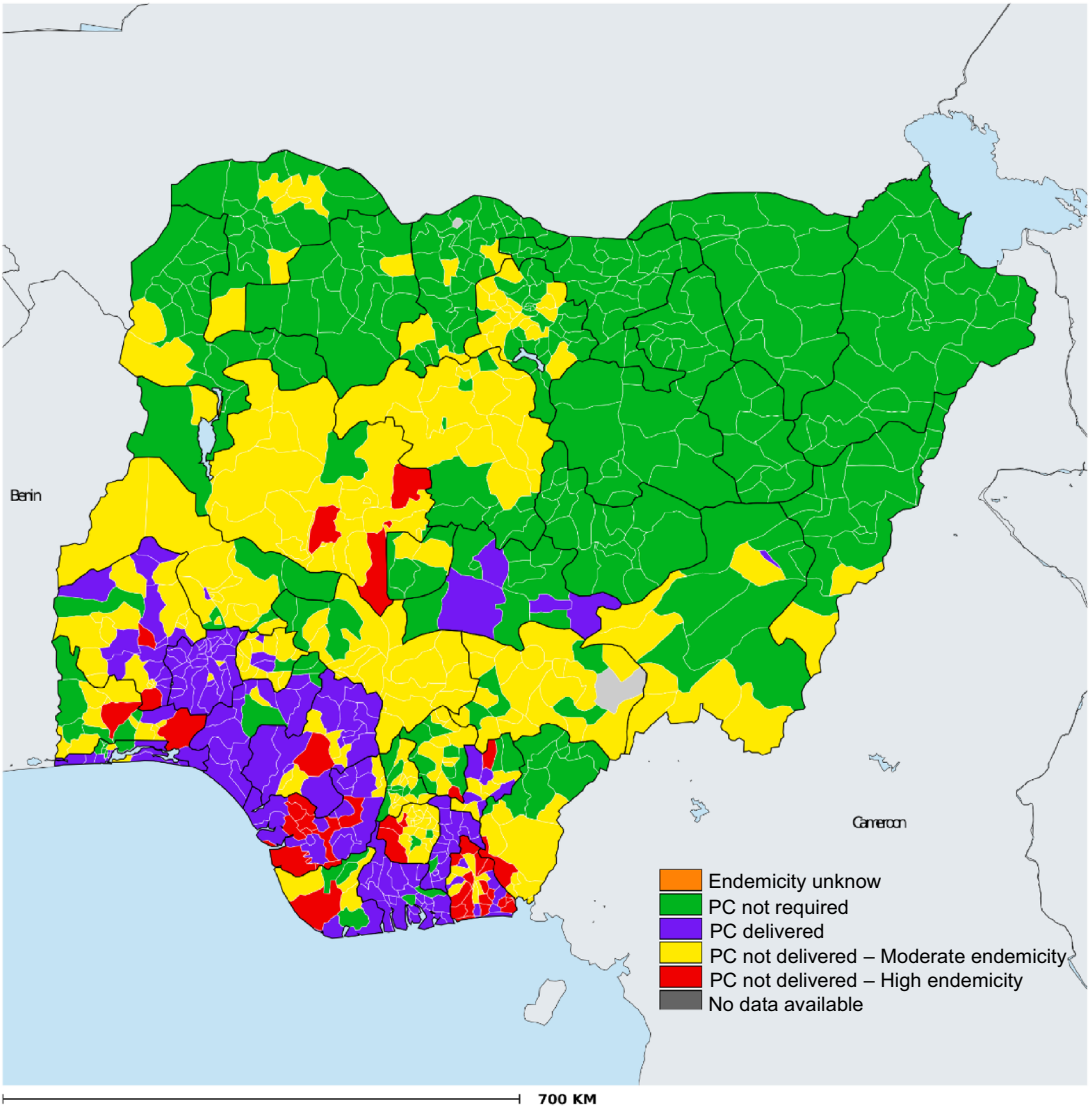
Geographical coverage—school-aged children STH preventive chemotherapy coverage in Nigeria—2021. *Data Source*: Data provided by health ministries to ESPEN through WHO reporting processes. All reasonable precautions have been taken to verify this information.

**Figure 3:**
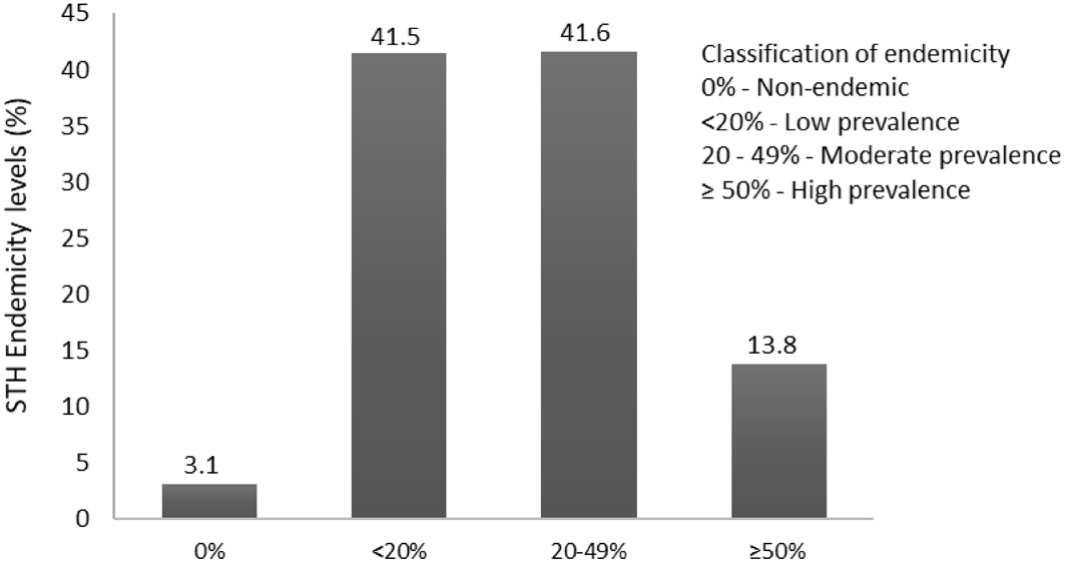
STH endemicity levels among Nigerian children.

The geographical distribution of STH preventive chemotherapy coverage among school-aged children in Nigeria is presented in Fig. 2. The map showed that PC was not delivered in several areas in the North-central, North-east and North-west of Nigeria. The percentage of children with STH relative to the endemicity level is presented in Fig. 3. There were no reported cases of STH among 3.1% of school-aged children. The percentages of children that harbour low, moderate, and high infections were 41.5, 41.6, and 13.4%, respectively (Fig. 3).

Of the 774 PC implementation units in Nigeria in the year 2021, PC was implemented in 177 units (22.9%) in school children. Effective (≥ 75%) PC implementation was achieved in 149 units (19.3%) (Table 1).

**Table 1:** The children’s proportion with preventive chemotherapy implementation.

Groups	PC implemented (%)	Effective (≥ 75%) PC implemented (%)
Yes	177 (22.9)	149 (19.3)
No	597 (77.1)	625 (80.7)
Total	774 (100)	774 (100)

*PC* preventive chemotherapy.

Table 2 showed the cumulative PC and effective PC implementation in the STH IUs in Nigeria in the year 2021. It was observed that PC was implemented only once between 2013 to 2021 among school children in 30(3.9%) IUs. Similarly, 43(5.6%) of the IUs implemented effective PC (≥ 75%) one time from 2013 to 2021. Generally, where interventions were implemented 1–4 times, more effective PC were implemented among school children in Nigeria. For example, the percentages of STH IUs where 1–4 interventions have been implemented between 2013 and 2021 ranged from 3.9 to 8.3% whereas the figures ranged from 5.6 to 21.3% where interventions were effectively implemented. Conversely, where the frequency of PC intervention ranged between 6 and 8 times, the percentages of STH IUs where effective PC was implemented drastically reduced (EffPC-n, 15.9–0.5%; PC-n, 23.6–6.1%) (Table 2).

**Table 2:** Cumulative PC implemented and effective PC implemented since 2013 based on programme coverage.

Treatment frequency (n)	Cumulative PC implemented since 2013 based on programme coverage—PC-n (%)	Cumulative Effective PC implemented since 2013 based on programme coverage—EffPC-n (%)
0	41 (5.3)	75 (9.7)
1	30 (3.9)	43 (5.6)
2	24 (3.1)	66 (8.5)
3	42 (5.4)	71 (9.2)
4	64 (8.3)	165 (21.3)
5	101 (13.0)	161 (20.8)
6	183 (23.6)	123 (15.9)
7	242 (31.3)	66 (8.5)
8	47 (6.1)	4 (0.5)
Total	774 (100)	774 (100)

The overall average PC coverage among school-aged children was 19.3%. Among STH low endemic population of children, PC coverage was 7.8% while it was 22.2% and 49.2% among children population with moderate and high infections, respectively (Table 3).

**Table 3:** STH endemicity and mean preventive chemotherapy coverage index in children.

Endemicity	Number of IUs	Mean ± SE	95% CI		*P* value
			Lower	Upper	
Low (≤ 20%)	321	7.8 ± 1.5	4.9	10.6	0.0001
Moderate (20–49%)	322	22.2 ± 2.2	19.9	26.6	
High (≥ 50%)	107	49.2 ± 4.3	40.7	57.7	
Total	774	19.3 ± 1.3	16.6	21.9	

*IUs* implementation units.

Table 4 showed the associations between PC intervention status and endemicity. The percentage of school-aged children with low STH prevalence was higher in the programme implementation units where PC was not implemented (49.2%) than in areas where STH PC was implemented (17.4%). However, school children had higher percentages of moderate and high STH endemicity where STH PC programme was implemented than where it was not implemented (*P* < 0.05). A similar trend was observed in areas where an effective STH PC programme was implemented compared to where it was not implemented (*P* < 0.05).

**Table 4:** Associations between different intervention indices and endemicity of STH in school-aged children.

Intervention indices	Endemicity				Total	*P* value
	Non-endemic	Low (≤ 20%)	Moderate (20%-49%)	High (≥ 50%)		
PC						
No	24 (4)	294 (49.2)	237 (39.7)	42 (7)	597 (100)	0.000
Yes	0 ()	27 (15.3)	85 (48)	65 (36.7)	177 (100)	
Total	24 (3.1)	321 (41.5)	322 (41.6)	107 (13.8)	774 (100)	
EffPC						
No	24 (3.8)	295 (47.2)	250 (40)	56 (9)	625 (100)	0.000
Yes	0 ()	26 (17.4)	72 (48.3)	51 (34.2)	149 (100)	
Total	24 (3.1)	321 (41.5)	322 (41.6)	107 (13.8)	774 (100)	
PC-n						
0	5 (12.2)	36 (87.8)	0 ()	0 ()	41 (100)	0.000
1	6 (20)	18 (60)	6 (20)	0 ()	30 (100)	
2	1 (4.2)	13 (54.2)	6 (25)	4 (16.7)	24 (100)	
3	0 ()	16 (38.1)	26 (61.9)	0 ()	42 (100)	
4	0 ()	12 (18.8)	23 (35.9)	29 (45.3)	64 (100)	
5	0 ()	34 (33.7)	51 (50.5)	16 (15.8)	101 (100)	
6	4 (2.2)	84 (45.9)	76 (41.5)	19 (10.4)	183 (100)	
7	8 (3.3)	97 (40.1)	112 (46.3)	25 (10.3)	242 (100)	
8	0 ()	11 (23.4)	22 (46.8)	14 (29.8)	47 (100)	
Total	24 (3.1)	321 (41.5)	322 (41.6)	107 (13.8)	774 (100)	
EffPC-n						
0	8 (10.7)	52 (69.3)	12 (16)	3 (4)	75 (100)	0.000
1	6 (14)	13 (30.2)	19 (44.2)	5 (11.6)	43 (100)	
2	1 (1.5)	21 (31.8)	34 (51.5)	10 (15.2)	66 (100)	
3	0 ()	23 (32.4)	29 (40.8)	19 (26.8)	71 (100)	
4	1 (0.6)	74 (44.8)	52 (31.5)	38 (23)	165 (100)	
5	4 (2.5)	61 (37.9)	85 (52.8)	11 (6.8)	161 (100)	
6	3 (2.4)	44 (35.8)	60 (48.8)	16 (13)	123 (100)	
7	1 (1.5)	30 (45.5)	30 (45.5)	5 (7.6)	66 (100)	
8	0 ()	3 (75)	1 (25)	0 ()	4 (100)	
Total	24 (3.1)	321 (41.5)	322 (41.6)	107 (13.8)	774 (100)	

*PC* preventive chemotherapy implemented, *PC-n* cumulative preventive chemotherapy implemented, *EffPC* effective preventive chemotherapy implemented, *EffPC-n* cumulative effective preventive chemotherapy implemented.

An association existed between the STH endemicity levels and the number of times PC was implemented (*P* < 0.05) (Table 4). The percentage of school-aged children with low STH prevalence was highest where PC programme was not implemented compared to where PC programme has been implemented once (60.0%), twice (54.2%), three times (38.1%), four times (18.8%) or eight times (23.4%). The highest percentage of school-aged children with moderate STH infection (61.9%) was recorded in IUs where PC was conducted three times whereas lower moderate infections were recorded where treatments were conducted twice (25.0%), four times (35.9%), five times (50.5%) or eight times (46.8%). Our results also showed that the percentage of high STH infection was highest (45.3%) in units where four treatment cycles were conducted when compared with units where treatments were conducted twice (16.7%), five times (15.8%), six times (10.4%) or eight times (29.8%) (Table 4).

The number of effective treatments (i.e. treatment coverage ≥ 75%) was significantly associated with STH endemicity levels (*P* < 0.05). The low endemicity of STH appeared to be generally higher where effective treatments were carried out 5 to 8 times compared to when carried out 1–3 times. Moderate STH prevalence was highest (52.8%) among school-aged children where effective treatments were conducted 5 times and lowest where there were no effective treatments (16.0%). Of the four implementation units where effective treatments were conducted 8 times, no high STH infection was recorded but 26.8% STH infection rate fell within the high endemicity level where effective treatments were conducted 3 times (Table 4).

## Methods

The year secondary data collected by the World Health Organization (WHO) through the expanded special project for elimination of neglected tropical diseases (ESPEN) and made available at https://espen.afro.who.int/tools-resources/download-data was used for the study. This data source is a robust digital platform for up-to-date data on five neglected tropical diseases (NTDs) in African countries. WHO Regional Office for Africa, African Member States, and NTDs Partners launched ESPEN in May 2016 in order to mobilize political, technical, and financial resources for the accelerated control and elimination of the five most common NTDs (lymphatic filariasis, onchocerciasis, schistosomiasis, soil-transmitted helminthiasis, and trachoma) with a focus in about 20 countries in Africa where NTDs are endemic. This collaboration with endemic countries has helped to reduce the cost of treatment for the above NTDs through resource mobilization. MDA entails administering medications to the entire population of a specified geographical area, regardless of the presence of symptoms or infection; however, exclusion criteria may apply. Assessment of the impact of the ESPEN partnership showed that 450 million of the 600 million people in Africa affected by NTDs every year have been saved until 2020. More than 600 people were trained to use ESPEN Collect, which synchronizes data with the ESPEN portal in about 20 countries and more than 13,300 sites^16^.

Available public data on the ESPEN portal are programmatic data shared by ministries of health from different countries. The data collected covered all of Nigeria's states, including the Federal Capital Territory (FCT), as well as 774 implementation units for each target population (Pre-SAC and SAC). The treatment indicators for STH and their measurements are presented below.

### Endemicity

This implies the reported STH endemicity status through the joint reported form (JRF) after final correction. It is measured on a 4-point scale rated as "0" (non-endemic), "1" [low prevalence (less than 20%)], "2" [moderate prevalence (20–49%)] "3" [high prevalence (50% and above)].

### Coverage (Cov)

This is defined as the ratio of total number of people treated with preventive chemotherapy (PC) to the total population targeted requiring PC. It is given as a percentage. The Pre-SAC stratum has a value for all of the implementation units (IUs) considered in the survey, indicating that no one in the target population received treatment, whereas the SAC stratum has values ranging from 0 to 127.50%, with an average of 19.27%.

### Preventive chemotherapy (PC)

It is a dichotomous variable that takes the value "1" if preventive chemotherapy is provided to the target population and "0" if PC is not provided to the target population.

### Effective PC (EffPC)

This is a binary variable that is coded as "1" if the PC implementation coverage in a specific target population is greater than 75% (effective), and "0" if the PC programme coverage is less than 75% (not effective).

### Cumulative PC implemented since 2013 based on programme coverage (PC-n)

This is a scale variable measured on a 9-point scale, with a score of "0" indicating that no PC was implemented in the target population from 2013 to 2021, and a score of "8" indicating continuous PC implementation in the target population from 2013 to 2021.

### Cumulative effective PC implemented since 2013 based on programme coverage (EffPC-n)

PC implementation in a target population is effective if the programme coverage is greater than 75%, and it is ineffective if the programme coverage is less than 75%. EffPC-n has values ranging from 0 to 8, with "0" indicating no effective PC implementation in the target population's implementation units (IUs), and "8" indicating consistent 9 years of effective PC implementation in the target population's IUs.

### Statistical analysis

The SPSS statistical package (version 25.0; IBM Corp., Armonk, NY, 2019) was used for all statistical analyses. The variables in the study were described using frequency counts, percentages, and charts. The association between endemicity and other categorical variables was examined using the chi-square test, however, one-way analysis of variance was used to check for variation of coverage across different endemicity statuses. Statistical significance was established for all hypotheses with *P* values less than 0.05.

## Discussion

Soil-transmitted helminthiasis was actively transmitted in all six geopolitical zones of Nigeria. The southern part of Nigeria was more endemic compared to northern Nigeria. There was no PC intervention in preschoolers and effective PC coverage fell below the WHO ≥ 75% PC coverage index benchmark in school children. PC was not delivered in several areas in the northern Nigeria. Multiple treatments did not necessarily reduce the endemicity of STH on certain occasions.

STH continues to be an important public health issue in several communities in Nigeria despite the renewed commitment of the global actors on NTDs control to eradicate these diseases by 2030. One of the major bottlenecks in addressing STHs (and other NTDs) in most endemic regions is delay in mapping the distribution of these diseases for planning effective preventive chemotherapy intervention. Over the years, PC implementation has been grossly skewed and sporadic, and therefore, has only produced little or no results in Nigeria. Recently, there is improvement in the coordination of STH PC implementation programmes, and the standard reporting system provided by the WHO makes it easy to track implementation progress.

ESPEN in partnership with the WHO developed a portal that enables health ministries and stakeholders to share, and exchange subnational programme data, in support of the NTDs’ control and elimination goals^17^. This development has become handy for easy tracking of endemic areas for monitoring implementation success and planning future programmes.

The distribution patterns of STHs in this study are similar to previous review work on the distribution of STHs across the six geopolitical zones of Nigeria^10^. The ongoing insecurity in Borno State, persisting for over a decade, could be the reason behind its non-endemic status. The region has faced exceptional challenges in implementing NTDs programmes. In fact, it remains the only state yet to complete baseline epidemiological mapping for certain PC-NTDs like onchocerciasis and lymphatic filariasis. Additionally, the relatively lower prevalence of STHs in other northern states may partly stem from limited research outputs, which could as well be linked to security challenges. Insufficient research outputs and lack of comprehensive baseline studies may have led to the incorrect categorization of many northern regions as areas requiring no intervention. Other factors responsible for the higher STH endemicity in the south compared to north could be attributed to the variations in environmental conditions such as temperature, humidity, rainfall, and soil moisture that stimulate environmental parasite egg development^18,19^. Hygiene levels and the extent of environmental contamination may be another factor^20^. The tropical rainforest feature of many places in the south, characterised by high rainfall, low humidity, and temperature^21^ can facilitate parasite egg development and dispersal, and increase the endemicity of STH as observed in the region. The high temperature, on the other hand, may contribute to lower STH infection status in northern Nigeria. High temperature has been reported to inactivate the eggs of *Ascaris*^22^, thus, reducing the transmission potency. The preponderance of a higher degree of open defecation in southern Nigeria compared to that of the north could be another contributing factor to the observed variations in STH endemicity^17^.

Although the STH endemicity pattern recorded in this study may not reflect the current endemicity level in Nigeria, the similar disease distribution pattern in preschool-aged and school-aged children is similar to a previous report in Kenya^23^. Despite the high endemicity of STH in the latter, and mounting evidence on the safety of the currently available deworming drugs, preschool children are consistently excluded from treatment, thus, posing a serious public health threat in the group. The WHO guidelines for STH recommend the treatment of preschool-aged children aged 1–5 years^24^. However, challenges such as the absence of a well-organized platform for intervention similar to school-based campaigns used for school-aged children intervention, inadequate and inconsistent availability of donated drugs, and the exclusion of preschool-aged children from lymphatic filariasis and onchocerciasis MDA programmes hinder their easy inclusion in routine PC-MDA.

Our results showed it is not uncommon to notice low PC coverage among children with low prevalence of STH, and a high percentage of children with low STH prevalence in areas where PC programmes have not been implemented. This is a typical occurrence since STH PC interventions primarily prioritize targeting the high infection groups in high-risk areas. The instances where higher levels of STH endemicity were observed after the implementation of PC, compared to areas where PC was not implemented, or when multiple rounds of treatment were administered, highlight the importance of integrating other interventions such as water, sanitation, and hygiene (WASH) with the STH PC programme in Nigeria. Poor sanitation characterised by open defecation and non-treatment of excreta before entering the environment facilitates STH transmission^25^. The worms’ high reproductive capability produces thousands of eggs, and therefore, are easily dispersed. Poor sanitation could stall a viable STH PC programme as this could promote reinfection and reversion of endemicity level to the pre-control status. Poor efficacy of the current STH regimens (albendazole and mebendazole) to hookworm and *Trichuris trichiura*^26^ could also be partly responsible for the persistent transmission of STH in Nigeria. The persistent transmission of STH in certain areas, despite the implementation of interventions, could potentially be attributed to instances of sabotage in the administration of PC interventions and the non-adherence of community-directed distributors to the WHO’s standard treatment recommendations for NTDs in the areas^27^.

It is essential to highlight that prevalence data in the ESPEN portal are typically obtained from diverse types of surveys, such as baseline mapping, impact assessments, and so on. These data are usually not collected in the same year due to various factors, resulting in as much as a 5-year gap between data in one IU and another, with varying approaches to design, sample collection, and parasite diagnosis. As a result, it becomes challenging to estimate the genuine level of STH endemicity among Nigerian children. Also, it is important to state that STH-PC intervention in Nigeria is not done in the entire 774 IUs. Thus, even if 75% PC coverage is met, it is difficult to evaluate PC coverage using the entire IUs. Notwithstanding, since the ESPEN data showed several endemic IUs but without intervention, and IUs without STH endemicity data, it is difficult to identify and separate IUs not to be included in the data analysis from the overall results.

In conclusion, this study revealed predominant moderate-to-high STH endemicity in southern Nigeria and low-to-moderate STH endemicity in northern Nigeria. Due to the absence of baseline data in the ESPEN data used, reaching a conclusive assessment on the substantial impact of PC in reducing STH transmission in Nigeria is challenging. Nonetheless, the presented data clearly demonstrates that STH remains endemic in the country. Currently, treatment is not effective as the country is far from realising the WHO ≥ 75% PC coverage. While the country should be more proactive and take ownership of the STH PC implementation programme, integration of WASH programme into the programme could produce a more viable outcome. The inclusion of preschool-aged children in PC implementation should be prioritised as they are at high risk and could serve as a reservoir of infection in the treated population.

## Supplementary Information

Supplementary Information.

## Data Availability

Data are available in the supplementary file provided.
